# A new negative feedback mechanism for MAPK pathway inactivation through Srk1 MAPKAP kinase

**DOI:** 10.1038/s41598-022-23970-8

**Published:** 2022-11-14

**Authors:** Maribel Marquina, Eva Lambea, Mercé Carmona, Marta Sánchez-Marinas, Sandra López-Aviles, José Ayte, Elena Hidalgo, Rosa Aligue

**Affiliations:** 1 Department of Biomedical Science, University of Barcelona, CIBERonc, Barcelona, Spain; 2 Department of Biosciences, Faculty of Mathematics and Natural Sciences, University of Oslo, Oslo, Norway; 3 Oxidative Stress and Cell Cycle Group, Universitat Pompeu Fabra, Barcelona, Spain; 4 Faculty of Health Sciences, Valencian International University (VIU), Valencia, Spain; 5 Present Address: Diagnostic Grifols SA., Barcelona, Spain; 6 Present Address: RPD. SL, Barcelona, Spain

**Keywords:** Cell biology, Molecular biology

## Abstract

The fission yeast mitogen-activated kinase (MAPK) Sty1 is essential for cell survival in response to different environmental insults. In unstimulated cells, Sty1 forms an inactive ternary cytoplasmatic complex with the MAPKK Wis1 and the MAPKAP kinase Srk1. Wis1 phosphorylates and activates Sty1, inducing the nuclear translocation of the complex. Once in the nucleus, Sty1 phosphorylates and activates Srk1, which in turns inhibits Cdc25 and cell cycle progression, before being degraded in a proteasome-dependent manner. In parallel, active nuclear Sty1 activates the transcription factor Atf1, which results in the expression of stress response genes including *pyp2* (a MAPK phosphatase) and *srk1*. Despite its essentiality in response to stress, persistent activation of the MAPK pathway can be deleterious and induces cell death. Thus, timely pathway inactivation is essential to ensure an appropriate response and cell viability. Here, uncover a role for the MAPKAP kinase Srk1 as an essential component of a negative feedback loop regulating the Sty1 pathway through phosphorylation and inhibition of the Wis1 MAPKK. This feedback regulation by a downstream kinase in the pathway highlights an additional mechanism for fine-tuning of MAPK signaling. Thus, our results indicate that Srk1 not only facilitates the adaptation to stress conditions by preventing cell cycle progression, but also plays an instrumental role regulating the upstream kinases in the stress MAPK pathway.

## Introduction

Eukaryotic cells have highly effective mechanisms for adapting to environmental changes that cause physiological stress. Exposure to environmental insults, such as high temperature, high osmolarity, genotoxic agents, or hydroxyl radicals, stimulates kinases to phosphorylate a variety of target substrates, including transcription factors that modulate gene expression^1–3^.

The fission yeast *Schizosaccharomyces pombe* MAPK Sty1, also called Spc1, is a stress-stimulated kinase essential for maintaining cell viability under diverse stressors^4–8^. Wis1, a MAPK kinase (MEK homolog), activates Sty1 through Thr-171 and Tyr-173 phosphorylation^4^. In *wis1∆* mutant cells, stress does not cause phosphorylation of Sty1^8–11^, indicating the involvement of no other MAPKK homologs in *S. pombe* Sty1 activation^12^. The activation of Sty1 by Wis1 is counteracted by Pyp1 and Pyp2 tyrosine phosphatases, with Pyp1 having the major activity during unperturbed vegetative growth^9^ and both being involved in MAPK dephosphorylation during stress^8^. Therefore, activity of the Sty1 MAPK is determined by a balance between Wis1 and both Pyp1 and Pyp2^8,13^. Disturbing this balance by either overexpressing *wis1* or simultaneously deleting *pyp1* and *pyp2* induces cytotoxic Sty1 hyperactivation^11,14^. Several Sty1 MAP kinase effectors have been identified, including the transcription factor Atf1, which is a homolog of mammalian ATF-2 and c-Jun^15–17^. In addition, Sty1 binds and phosphorylates MAPK-activated protein kinases (MAPKAP), such as Srk1 (Sty1-regulated kinase), that are also related to mammalian calmodulin-dependent kinases^18^. Srk1 regulates the onset of mitosis by phosphorylating and inhibiting the phosphatase Cdc25 response to non-genotoxic osmotic or oxidative stress^18–20^.

In this work, we provide evidence that, after stress-induced activation by Sty1, Srk1 phosphorylates the MAPKK Wis1 to reduce MAPK activity and regulate the stress response. This novel feedback mechanism represents an additional layer of control contributing to the timely and robust inactivation of the stress signaling pathway.

## Results

### Absence of Srk1 leads to Sty1 hyperactivation

**Figure 1:**
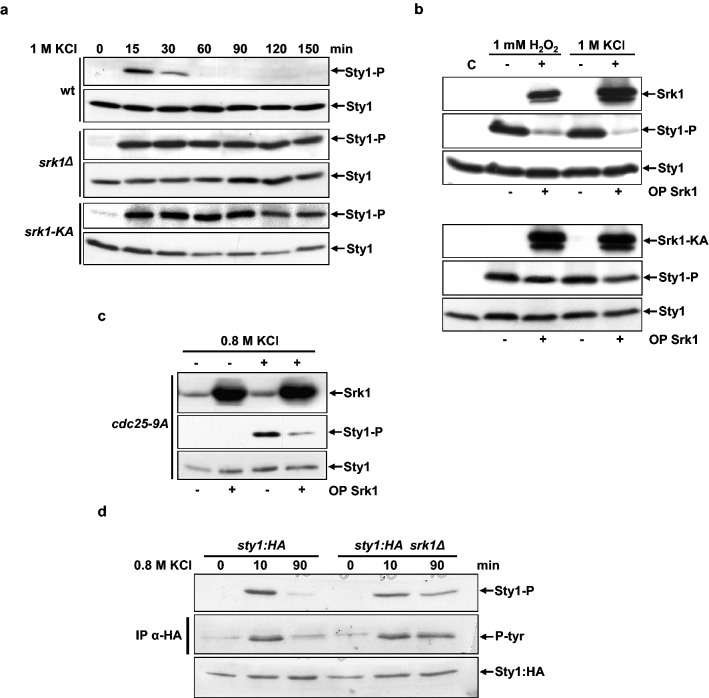
Srk1 kinase is involved in Sty1 inactivation after stress response. (**a**) Total extracts from growing cultures of wild-type, *srk1Δ* and *srk1-KA (catalytically inactive srk1)* strains, either untreated (0 min) or treated with 1 M KCl at the indicated times, were resolved in SDS-PAGE and analyzed by western blotting. Blot membranes were cut prior antibodies incubation, Activated Sry1 (Sty1-P), corresponding to Sty1 phosphorylated at Thr171 and Tyr173 was detected with an anti-phospho p38 antibody and total Sty1 protein was detected with anti-HOG1 antibody. (**b**) Srk1 overexpressed (+ OP Srk1) through the activation of the inducible *nmt* promoter of the plasmid *pREP1-srk1:HA6his* in the absence of thiamine (−B1) during 20 h or repressed (−OP Srk1) by adding thiamine (*pREP-srk1:HA6his* + B1) in wild-type cells, untreated or treated with 1 mM H_2_O_2_ and 1 M KCl for 15 min. Activated (Sty1-P) and total Sty1 were detected with anti-phospho p38 and anti-HOG1 antibodies, respectively. Level of overexpressed Srk1 was detected with anti-HA antibody. Lane C, indicates a control which correspond to untreated wild-type cells. (**c**) *cdc25-9A* cells with (+) or without (−) Srk1 overexpression for 20 h (OP Srk1: *pREP1-srk1:HA6his –B1*) and untreated or treated with 0.8 M KCl for 15 min. Activated and total Sty1 were detected with anti-phospho p38 and anti-HOG1 antibodies, respectively. Level of overexpressed Srk1 was detected with anti-HA antibody. (d) *S. pombe* wild-type and *Δsrk1* cells expressing genomic *sty1:HA* were untreated (0 min) or treated with 0.8 M KCl at the indicated times (min). Activated and total Sty1 were detected with anti-phospho p38 and anti-HA antibodies, respectively. Sty1:HA was immunoprecipitated from each time point, and Sty1 tyrosine phosphorylation was detected with anti-phospho-tyrosine antibody (middle panel). Blot membranes were cut prior antibodies incubation.

Stress conditions transiently activate the MAPK Sty1. Fission yeast cells exposed to 1 M KCl depict maximal Sty1 phosphorylation after 15–20 min of treatment. Phosphorylation starts, decreasing after 30 min, and is no longer detectable after 60 min of stress exposure (Fig. 1a, upper panel). Strikingly, under the same stress conditions (1 M KCl) deletion of *srk1* led to sustained activation of Sty1 (Fig. 1a, middle panel). This result was further confirmed using a mutant allele of *srk1* (*srk1-K153A,* hereafter referred to as *srk1KA*), expressing catalytically inactive Srk1. Similar to *srk1∆*, the *srk1-KA* mutant failed to inactivate Sty1 and phosphorylation was still observable after 150 min of osmotic stress exposure (Fig. 1a, lower panel). These results point towards the involvement of Srk1 in Sty1 inactivation.

To better characterize this effect, we next overexpressed Srk1 and exposed cells to either osmotic stress (1 M KCl) or oxidative stress (1 mM H_2_O_2_). Under both conditions overexpression of Srk1 resulted in a reduction of Sty1 phosphorylation (Fig. 1b). By contrast, overexpression of Srk1-KA under the same stress conditions did not produce significant changes in Sty1 phosphorylation compared to control cells (Fig. 1b), confirming the negative effect of Srk1 in Sty1 activity.

Activation of Srk1 by osmotic and oxidative stress induces cell cycle arrest through Cdc25 phosphorylation. To explore whether Cdc25 mediates the negative effect of Srk1 over Sty1, we overexpressed Srk1 in cells where the *cdc25* gene had been replaced by a mutant allele containing alanine substitutions for 9 Srk1 phosphorylation sites (*cdc25-9A*). In this genetic background Srk1 can no longer inhibit Cdc25 and prevent cell cycle progression^18,20^. Nevertheless, as shown in Fig. 1c, Srk1 overexpression still inhibited Sty1 phosphorylation in response to osmotic stress. Thus, the role of Srk1 in regulating Sty1 activation was considered independent of Cdc25 and the regulation of cell cycle progression.

Wis1 activates Sty1 by phosphorylating it at two sites, threonine 171 (T171) and tyrosine 173 (Y173). The antibody against phosphorylated Sty1 recognizes both T171 and Y173, so the site affected by the absence of *srk1* is unknown. In addition, the phosphorylation of Y173 is essential for Sty1 activity^21^. Therefore, we analyzed Sty1 phosphorylation of tyrosine in *srk1*-deleted cells. Sty1 fused to the HA epitope was immunoprecipitated from wild-type and *srk1∆* cells treated with 0.8 M KCl, and tyrosine phosphorylation was examined by western blot with an anti-phospho-tyrosine antibody (Fig. 1d). The result showed clearly enhanced Sty1-tyrosine phosphorylation in *srk1∆* cells exposed to stress (Fig. 1d).

### The effect of Srk1 on Sty1 activation is independent of Pyp1 and Pyp2 phosphatases

**Figure 2:**
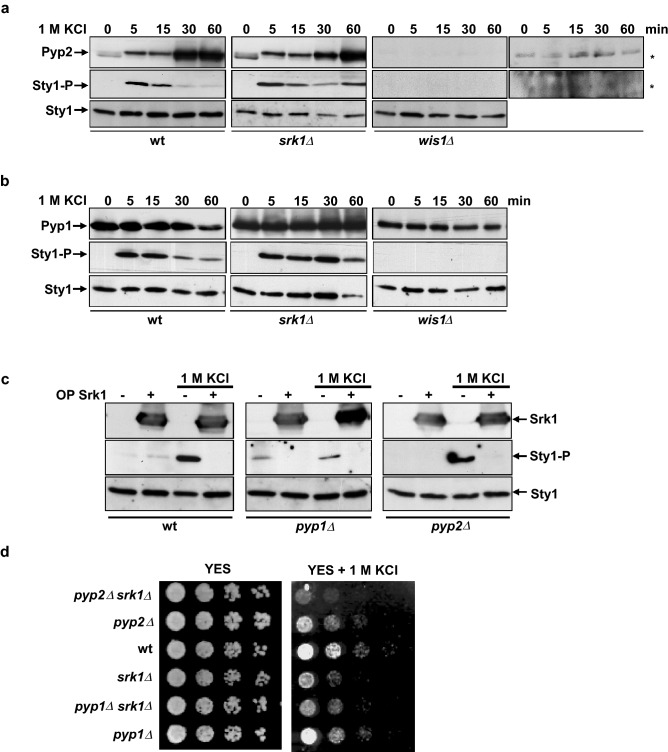
MAP phosphatase levels and activity are not dependent on Srk1. (**a**) and (**b**), *S. pombe* wild-type, *srk1∆,* and *wis1∆* cells expressing genomic *pyp2:HA6his* (**a**) and *pyp1:HA6his* (**b**) were grown in YES medium to mid-log phase and remained untreated (0 min) or treated with 1 M KCl at the indicated times, up to 60 min. Activated (Sty1-P) and total Sty1 were detected with anti-phospho p38 and anti-HOG1 antibodies, respectively. Total Pyp2 (**a**) and Pyp1 (**b**) levels were detected with anti-HA antibody. *longer exposure of Pyp2 and P-Sty1 levels are shown in *Δwis1.* (**c**) Wild-type, *pyp1∆* and *pyp2∆* cells with ( +) or without (-) Srk1 overexpression (OP Srk1: through the inducible plasmid *pREP1-srk1:HA6his)* during 20 h, were untreated (0 min) or treated with 1 M KCl for 15 min. Activated (Sty1-P) and total Sty1 were detected with anti-phospho p38 and anti-HOG1 antibodies, respectively. The level of overexpressed Srk1 was detected with anti-HA antibody. All blot membranes were cut prior antibodies incubation, (**d**) Decimal dilutions of strains of the indicated phenotypes were spotted on YES solid plates with or without 1 M KCl that had been incubated at 30 °C for 3 days and photographed.

As mentioned above, Pyp1 and Pyp2 tyrosine phosphatases counteract Sty1 activation, with Pyp1 having a more prevalent role^9^. Therefore, given our observations, we next considered whether the role of Srk1 precluding Sty1 activity was mediated by these phosphatases. To check this, we first determined the levels of Pyp1 and Pyp2 in *srk1∆* and wild-type cells. As Fig. 2 shows, the levels of Pyp1 and Pyp2 were comparable in both strains (Fig. 2a and b), indicating that Srk1 does not regulate the levels of these phosphatases .This experiment also indicated that the sustained phosphorylation of Sty1 that we observed in *srk1∆* cells represents the active form of MAPK, since *pyp2* gene expression is fully dependent on Sty1 activation in response to stress^15,22^ (Fig. 2a). In contrast, when the same experiment was performed in *wis1∆*, Sty1 did not become phosphorylated in response to stress and Pyp2 was absent (Fig. 2a). For Pyp1, the phosphatase exhibited constitutive expression independent of Sty1 activation, as previously described^9^ (Fig. 2b).

Having shown that Srk1 does not regulate Pyp1 or Pyp2 protein levels, we then considered whether it could regulate their activity. If that was the case, one would expect that the reduction of Sty1 phosphorylation following Srk1 overexpression would be abolished in the absence of the phosphatases. However, as shown in Fig. 2c, deletion of either *pyp1* or *pyp2* did not restore Sty1 phosphorylation levels in cells overexpressing Srk1 and exposed to osmotic stress. Of note, basal Sty1 activation occurred in *pyp1∆* cells (Fig. 2c), which is coherent given that basal Pyp1 levels maintain inactive Sty1 under normal conditions.

These results suggested that Srk1 does not exert its role in the regulation of the SAPK pathway through Pyp1 and Pyp2 phosphatases. This idea was further confirmed when we analyzed the sensitivity of single (*srk1∆, pyp1∆* and *pyp2∆*) and double mutants (*srk1∆ pyp1∆* and *srk1∆ pyp2∆*) to osmotic stress. The sensitivity of double mutant was greater than that of single mutants (Fig. 2d), confirming that the negative regulation of the SAPK pathway by Srk1 and the phosphatases is additive.

### Atf1 transcriptional response is independent of Srk1

**Figure 3:**
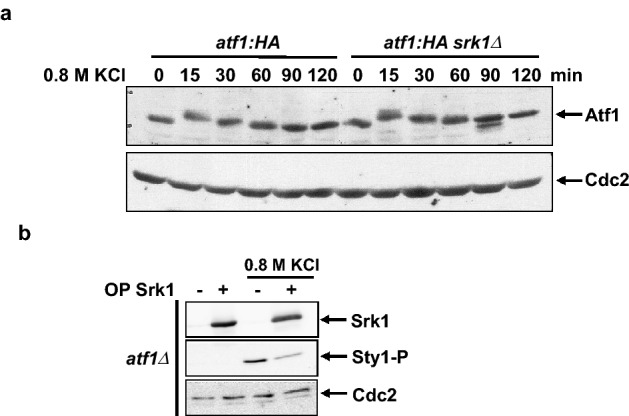
Atf1 response to stress is independent of Srk1. (**a**) Total extracts from growing cultures of wild-type and *srk1∆* cells expressing genomic *atf1:HA6his* were untreated (0 min) or treated with 0.8 M KCl at the indicated times and resolved in SDS-PAGE. Total Atf1 protein was detected with anti-HA antibody. (**b**) *atf1* deleted cells with (+) or without (−) Srk1 overexpression (OP Srk1: through the inducible plasmid *pREP1-srk1:HA6his*) for 20 h were untreated or treated with 0.8 M KCl for 15 min. Activated (Sty1-P) and total Sty1 were detected with anti-phospho p38 and anti-HOG1 antibodies, respectively. The level of overexpressed Srk1 was detected with anti-HA antibody. Anti-Cdc2 was used as a loading control (**a** and **b**). All blot membranes were cut prior antibodies incubation.

The delay in Sty1 dephosphorylation after stress in *∆srk1* could result from a defect in the adaptive response; thus, cells would need more time to adjust to the new conditions. Since the transcriptional changes induced by Sty1-dependent transcription factor Atf1 represent the main mechanism of stress adaptation, we set out to evaluate the involvement of Srk1 in this process. We examined the protein levels of Atf1 in wild-type and *∆srk1* cells after KCl treatment. As Fig. 3a shows, *srk1* deletion did not produce significant changes in Atf1 levels in response to osmotic stress. In parallel, we also overexpressed Srk1 in *atf1∆* cells to assess whether in the absence of the transcription factor (i.e., without the induction of the *CESR –Core Environmental Stress Response-* genes), Srk1 could prevent Sty1 phosphorylation induced by KCl. Again, the inhibitory effect of Srk1 was not affected by the absence of Atf1 (Fig. 3b), indicating that it is independent of the induction of the adaptive transcriptional program.

### Sty1 stays in the nucleus in the absence of Srk1

**Figure 4:**
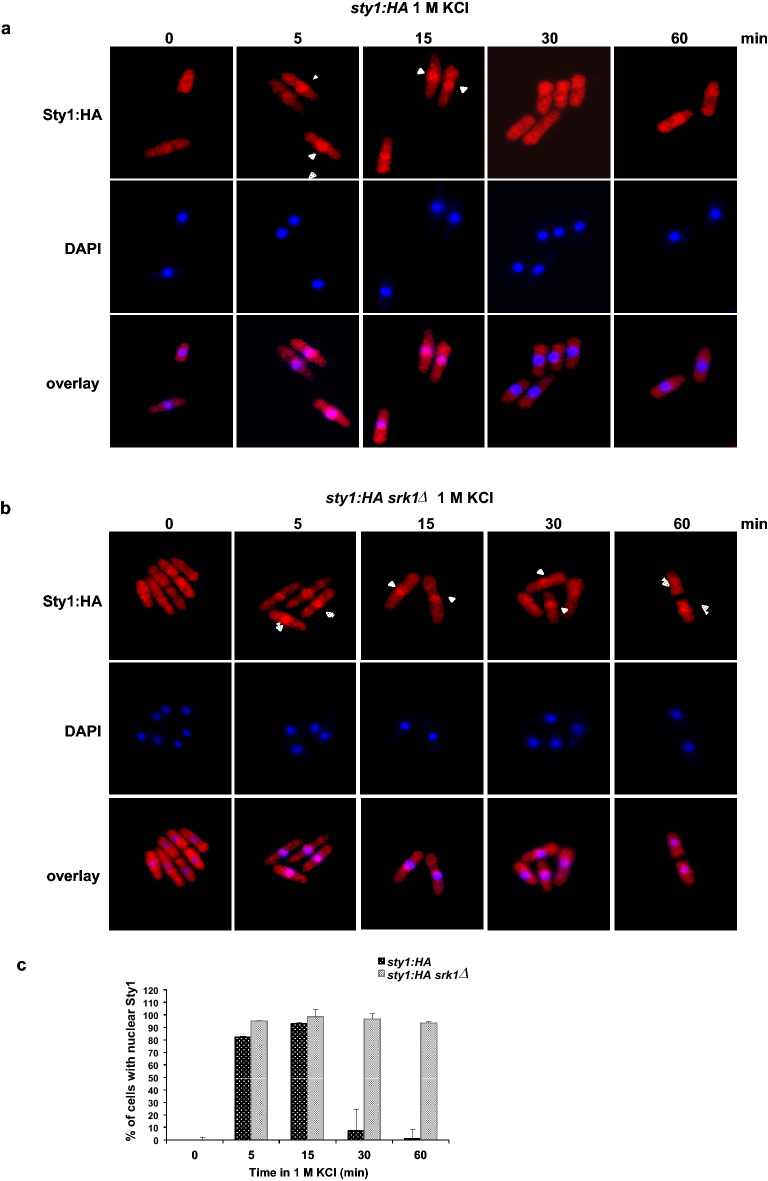
Sty1 localization in response to stress is altered in absence of Srk1. Wild-type (**a**) and *srk1∆* (**b**) cells expressing genomic *sty1:HA* were grown in YES medium to mid-log phase, remained untreated (0 min) or treated with 1 M KCl, and fixed with methanol at the indicated times. Sty1 localization was detected by indirect immunocytochemistry with anti-HA primary antibody (upper panels) and nuclei were stained with DAPI (middle panels). An overlay of both is shown in the lower panels. (**c**) Cells with Sty1 localized in the nucleus, in *sty1:HA* and *sty1:HA srk1*Δ cells treated with 1 M KCl at indicated times, were quantified. Data represent the average of two biological replicates.

We then addressed whether Srk1 affects Sty1 cell localization by subjecting wild-type and *srk1∆* cells expressing HA-tagged Sty1 to osmotic stress and performing indirect immunocytochemistry. As already described^23^, Sty1 rapidly translocated to the nucleus in response to 1 M KCl (5 min) and returned to the cytoplasm after 30 min (Fig. 4a) where it is inhibited by the phosphatases. In contrast, although we observed Sty1’s swift nuclear translocation 5 min after exposing *srk1∆* cells to 1 M KCl the MAPK had not gone back to the cytoplasm after 60 min of treatment (Fig. 4b and c). This suggested that Srk1 is required for proper Sty1 localization in response to stress.

### Srk1 phosphorylates Wis1

**Figure 5:**
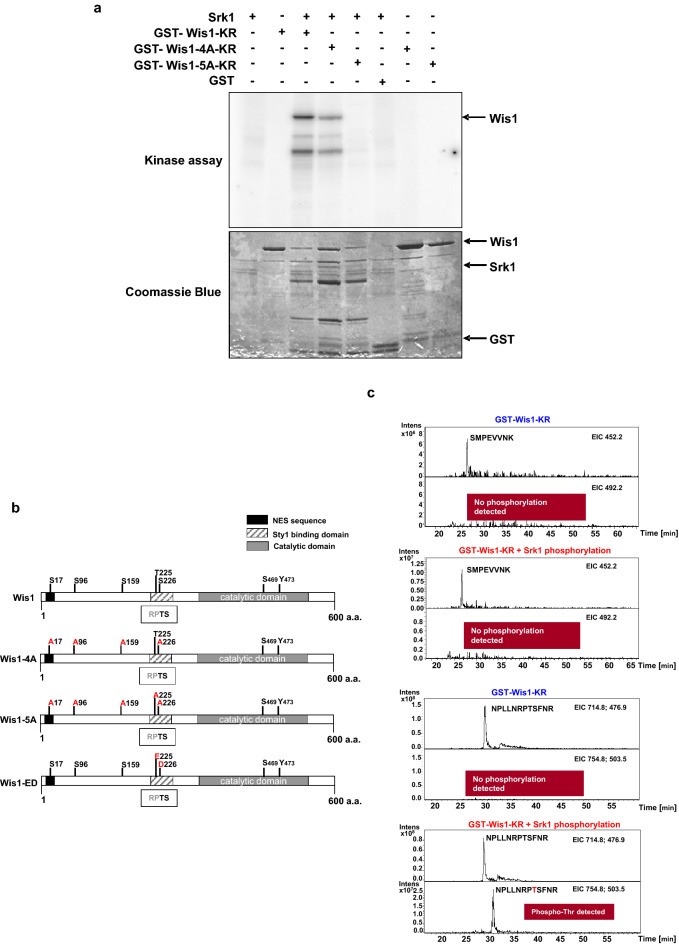
Wis1 is phosphorylated by Srk1 (**a**) Recombinant Srk1, GST-tagged Wis1-KR (catalytically inactive) Wis1-4A-KR, and Wis1-5A-KR were incubated separately or together with γ^32^P-ATP in the absence or presence of recombinant Srk1. The autoradiogram (top) and Coomassie blue (bottom) results are shown. (**b**) Schematic representation of Wis1 protein domains and the different mutations performed to obtain Wis1 phospho-mutants (see text for details). (**c**) In vitro phosphorylation assays with recombinant GST-Wis1-KR without (blue planels) or with recombinant GST-Srk1 (red panels) and processed for LC–MS phosphopeptide analysis. Two peptides are shown, peptide SMPVVNK (159–166 AA) (first two panels) and peptide NPLLNRPTSFNR (218–229 AA) (last two panels).

Considering the above results, we hypothesized that Srk1 modulates Sty1 activity via Wis1, presumably through inhibitory phosphorylation of the MAPKK. To identify Wis1 as a direct substrate of Srk1, we performed an in vitro kinase assay using recombinant Srk1 as the kinase and a recombinant catalytically inactive Wis1 (GST-Wis1-K349R or GST-Wis1-KR) as the substrate. Confirming our idea, Srk1 could efficiently phosphorylated Wis1 in vitro (Fig. 5a, tree first lanes).

Next, we visually scanned the Wis1 protein sequence to identify potential phosphorylation consensus residues (RXXS/T) for Srk1 and found four such residues: Ser17 (within the NES sequence), Ser96, Ser159, and Ser226 (within the Sty1 binding domain) (Fig. 5b and Supplementary information Fig. S1). We mutated all four serine residues to alanine and expressed the resulting mutant as recombinant GST-Wis1-4A-KR, in order to test its phosphorylation by Srk1 in vitro (Fig. 5a). Surprisingly, mutation of the four consensus sites attenuated but did not completely abolish Wis1 phosphorylation by Srk1 (Fig. 5a). This led us to think that Srk1 could be phosphorylating a non-consensus residue within the Wis1 sequence. To answer this question, we carried out a phosphoproteomic analysis of in vitro phosphorylated GST-Wis1-KR. Strikingly, a new non-consensus residue, T225 adjacent to S226, was detected in the Wis1 sequence as being phosphorylated by Srk1 (Fig. 5c and b).

To validate this result, we mutated T225 to Ala in our recombinant GST-Wis1-4A-KR construct, thus generating a new GST-Wis1-5A-KR construct (Fig. 5b), that we tested in, in vitro kinase assay. As Fig. 5a shows, mutation virtually abrogated Wis1 remaining phosphorylation, confirming that Srk1 phosphorylated Wis1 mainly in T225.

### Srk1 phosphorylation inhibits binding between Wis1 and Sty1

**Figure 6:**
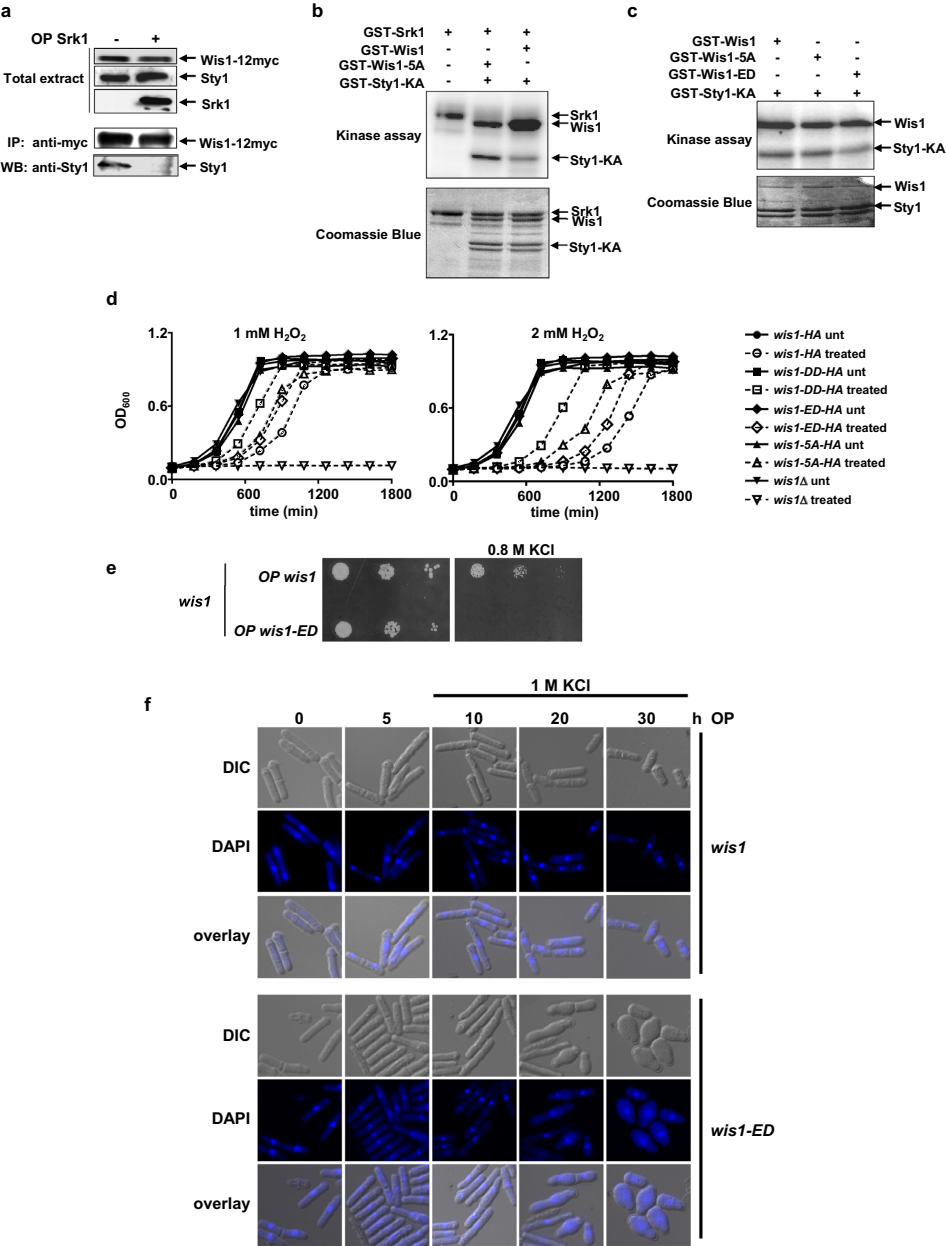
Srk1 activity decreases Wis1 binding and Sty1 activation. (**a**) Wis1:12myc was immunoprecipitated using anti-myc antibody, from cells with (+) or without (−) Srk1 overexpression (OP Srk1) for 20 h containing genomic *wis1:12myc*. Sty1 binding was detected with anti-HOG. Total Wis:12myc, Sty1, and Srk1 levels were detected by western blot with anti-myc, anti-HOG, and anti-HA antibodies from total extracts of the same samples, respectively. Blot membranes were cut prior antibodies incubation, (**b**) In vitro kinase assay with recombinant GST-tagged Wis1, Wis1-5A and Sty1-KA (catalytically inactive) were incubated with γ^32^P-ATP and recombinant GST-Srk1. The autoradiogram (top) and Coomassie blue (bottom) results are shown. (**c**) In vitro kinase assay with recombinant GST-tagged Wis1, Wis1-5A, and Wis1-ED were incubated with γ^32^P-ATP and recombinant GST-Sty1-KA (catalytically inactive). The autoradiogram (top) and Coomassie blue (bottom) results are shown. (**d**) Growth curves of wild-type, Wis1-DD, Wis1-5A, Wis1-ED and *wis1∆* cells growing in MM medium plus the indicated concentrations of H_2_O_2_. The graph represents the more representative of three biological replicates. (**e**) Decimal dilutions of *wis1*-deleted cells overexpressing either *wis1* (pREP81-*wis1 *–B1) or *wis1-ED* (pREP81-*wis-ED* –B1) were spotted on YES solid plates with or without 0.8 M KCl, incubated at 30 °C for 3 days, and photographed. (**f**) *wis1*-deleted cells overexpressing either *wis1* (pREP81*-wis1* –B1) or *wis1-ED* (pREP81*-wis-ED* –B1) were treated with 1 M KCl after 10 h of overexpression. Cells were stained with DAPI at the indicated times and observed in a microscope (objective ×63).

As indicated in Fig. 5b, residues T225 and S226 lie within the binding domain for the MAPK Sty1 (200–300 AA)^24^. Hence, we envisioned that Srk1 could inhibit binding between Wis1 and Sty1 and thus prevent pathway activation. A first approach to confirm this was to overexpress Srk1 in *wis1-12myc ∆srk1* cells and study the binding between Wis1 and Sty1 by immunoprecipitation and western blot. Figure 6a shows that Srk1 overexpression completely eliminated binding between Wis1 and Sty1, suggesting that it may phosphorylate T225 in vivo to inhibit the binding between these kinases and thereby silence the pathway.

Next, we studied the effect of Srk1 phosphorylation on Wis1 activity towards Sty1. We performed an in vitro kinase assay with recombinant Wis1 and Wis1-5A (GST-Wis1 and GST-Wis1-5A, respectively) that had been previously incubated with recombinant Srk1 (GST-Srk1), and added these to catalytically inactive recombinant Sty1 (GST-Sty1-KA). Wild type Wis1 phosphorylated Sty1 to a lesser extent than Wis1-5A (Fig. 6b) which correlated with the inability of Srk1 to phosphorylate Wis1-5A and the higher affinity of Wis1-5A for Sty1. To confirm the critical role of Wis1 phosphorylation, we extended our analysis to a Wis1 phospho-mimetic mutant (GST-Wis1-ED) in which T225 and S226 were mutated to glutamic and aspartic acid (E225 and D226) to mimic threonine and serine phosphorylation states, respectively (Fig. 5b). Both Wis1-5A and Wis1-ED phosphorylated Sty1-KA (Fig. 6c), but phosphorylation by Wis1-ED was slightly reduced compared to Wis1 and Wis1-5A (Fig. 6c). This could be consistent with Wis1-ED having a lower affinity for Sty1, and therefore, an impaired capacity for Sty1 activation.

In order to confirm the in vivo relevance of Wis1 phosphorylation by Srk1 in the regulation of the MAPK pathway, we analyzed the growth of strains where endogenous *wis1* had been replaced by either the *wis1-5A* or *wis1-ED* alleles. Strains expressing HA-tagged *wis1* or the constitutively active *wis1-DD* allele (containing Asp substitutions at the MAPKKK phosphorylation sites) were used as control. As expected, stress exposure induced a significant delay in the proliferation of wild type cells. In contrast, in the *wis1DD* mutant this delay was less pronounced and cells resumed proliferation shortly after the treatment. Albeit not to the same degree, exchange of *wis1* with the phospho-null *wis1-5A* allele also attenuated the delay following stress exposure (Fig. 6d). Lastly, in the case of cells carrying the *wis1-ED* allele (mimicking the Srk1-inactivated form of Wis1) growth was slower than for the *wis1-5A* mutant (Fig. 6d).

The quantification of these changes is summarized in Table 1. We considered the delay in growth resumption experienced by wild type cells exposed to stress compared to mock-treated cells to be 100%. While *wis1-DD* cells only display a 30% delay, *wis1-5A* cells exhibit a 50% growth delay versus wild type cells (Table 1). Altogether,these results indicate a clear inhibitory effect on MAPK signaling mediated by the phosphorylation of Wis1 by Srk1.

**Table 1:** The delay time of untreated and H_2_O_2_–treated growth curves, determined at an apparent OD_600_ of 0.5, is shown in minutes (min) and percentage (%). Standard deviation from biological triplicates is indicated.

Strain	1 mMH_2_O_2_		2 mMH_2_O_2_	
	Delay time (in min)	Delay time (in %)	Delay time (in min)	Delay time (in %)
*wis1-HA*	408 ± 46	100	982 ± 99	100
*wis1.DD-HA*	130 ± 13	32	285 ± 66	29
*wis1.5A-HA*	217 ± 29	53	532 ± 54	54
*wis1Δ*	NG^a^	NG	NG	NG

^a^No growth detected for H_2_O_2_–treated cultures.

We then determined if Wis1 phosphorylation by Srk1 affected the response to stress by expressing wild type *wis1* or the *wis1-ED* allele at an endogenous level, under the control of the weakest version of the *nmt*-promoter (*nmt81*), in *wis1∆* cells. Cells were exposed to osmotic stress and their survival was analyzed using a spot dilution assay. As Fig. 6e shows, the expression of wild-type *wis1* allowed *wis1∆* cells to respond to stress, while the expression of *wis1-ED* did not sustain a robust stress response.

In addition, to directly test how cells respond to osmotic stress, *wis1∆* cells expressing *wis1* or *wis1-ED* under control of the *nmt81* promoter for 10 h (to allow sufficient expression) were exposed to KCl and samples were taken at intervals up to 30 h (Fig. 6f). As expected, *wis1∆* cells expressing *wis1* responded to stress and showed a wild-type phenotype. By contrast, *wis1∆* cells expressing Wis1-ED showed defects in morphology and exhibited an aberrant phenotype (Fig. 6f). These results demonstrate that constitutive phosphorylation of Wis1 by Srk1 (Wis1-ED) disregulates the MAPK response.

## Discussion

In this paper, we have presented evidence that describe a new role of Srk1 as a negative regulator of the Sty1 MAPK pathway. We concluded that this occurred by inhibiting Wis1 through direct phosphorylation in the Sty1 binding domain. Srk1 phosphorylation reduced the binding between Wis1 and Sty1, allowing subsequent inactivation of the pathway by the Sty1 phosphatases Pyp1 and Pyp2. The observation that the MAPK Sty1 is phosphorylated in *srk1∆* cells exposed to stress over time, unlike wild-type cells that displayed a sharp peak in activation, supports our conclusion. Rather than this being a side effect of the deletion, the lack of Srk1 kinase activity is likely the cause of the sustained activation of Sty1, since using catalytically inactive *srk1-KA* produced the same effect as *srk1* deletion.

Signaling through the MAP kinase pathway involves the activation of different mechanisms in response to environmental stress, but once the source of stress has disappeared or the cell has adapted to it, proper inactivation of the MAPK pathway is essential^8,13^. Since pathway activation involves a series of successive phosphorylations, these will have to be reversed by the action of phosphatases involved in this process. For *S. pombe*, Pyp1 and Pyp2 phosphatases bind and dephosphorylate Tyr173 from Sty1 in response to stress, leading to the inactivation of the pathway^8,11,22^.

As a first step in understanding Srk1's negative feedback, we hypothesized that it might act by enhancing the expression of Pyp1 and Pyp2 tyrosine phosphatases. However, analysis of Pyp1 and Pyp2 protein levels in the wild-type and *srk1∆* strains revealed no change in their protein levels. Yet, this experiment showed that sustained phosphorylation of Sty1 in *srk1∆* cells correlates with the active form of the MAPK, due to the dependence on Sty1 activation to stimulate Pyp2 expression in response to stress^22^. We also discarded the idea that Srk1 could modulate the activity of these phosphatases, since Srk1 overexpression still led to dephosphorylation of Sty1 in *pyp1∆* or *pyp2∆* cells. Supporting these results, a stress sensitivity analysis of double *srk1∆ and* phosphatase mutants indicated that Srk1 and the two phosphatases act independently. Thereafter, we focused on the MAPKK Wis1 as a putative substrate of Srk1. Some observations have suggested that Wis1 is phosphorylated at residues other than activation residues Ser469 and Thr473 in response to stress^5^.

Notably, although Wis1 has four potential phosphorylation consensus motifs (RXXS/T) for Srk1 in its regulatory domain, mutation of these residues to Ala reduced its phosphorylation by Srk1 only marginally. Nevertheless, phosphopeptide analysis identified T225 as a non-consensus site phosphorylated by Srk1, which is located within the Wis1-Sty1 binding domain. Several evidences have indicated that T225 is the main Wis1-phosphorylated site by Srk1 involved in the negative regulation of the pathway. In vitro kinase assays showed that Wis1 preincubated with Srk1 has a lower affinity for Sty1 than the Wis1-5A treated in the same manner, resulting in lower activity. More importantly, growth curves of *wis1-5A* cells compared to wild type and *wis1-DD* (constitutively active Wis1 mutant) cells as control, confirm that phosphorylation of Wis1 by Srk1 is involved in the negative regulation of MAPK pathway after the stress response. In contrast, the growth of *wis1-ED* cells exposed to stress was reduced compared to *wis1-5A* mutant, as expected given the reduced phosphorylation of Sty1 in this mutant; however, *wis1-ED* cells were still capable of activating the pathway since their growth was indeed greater than that of wild-type cells. Still, cells expressing this mutant allele and exposed to stress lost their viability in the long term. This suggests that, while reduced MAPK pathway activation attenuates the cell cycle arrest in response to stress, it is deleterious for the survival of the cell when stress conditions persist for longer periods.

One question derived from the experiment presented here is, why is it necessary inhibiting the MAPKK Wis1 in addition to the well described inhibition of Sty1 by phosphatases?

The latter mechanism is in principle sufficient to inhibit the pathway after a stress insult. However Sty1 regulation has important several characteristics. Upon phosphorylation and activation by Wis1, at T171 and Y173, Sty1 moves to the nucleus where it triggers the transcription of several genes instrumental to the stress response. Among them, the phosphatases Pyp1 and Pyp2, and the kinase Srk1. During the stress response, Sty1 actively shuttles from de nucleus to the cytoplasm^23^ and vice versa, until it becomes dephosphorylated by Pyp1 and Pyp2 in the cytoplasm. If the stress signal does not diminish, the cytoplasmic inactive Sty1 can be phosphorylated again by Wis1 and promote a sustained toxic stress response. As for Sty1, hyperactivation by means of overexpression of Wis1 is toxic to wild-type cells and inhibits colony formation, even though these cells express the counteracting phosphatases Pyp1 and Pyp2^13,25^. Therefore, it is reasonable to think that additional steps regulating the upstream kinase in the pathway are required to ensure the inactivation of the MAPK and to avoid toxicity.

Our Sty1 localization results also support the hypothesis that Srk1 inhibits Wis1 in parallel to Sty1 de-phosphorylation by Pyp1 and Pyp2 to preclude the activity of the pathway. Whereas, in wild-type cells exposed to stress, Sty1 nuclear activity was time-limited (from 5 to 30 min), in the *srk1∆* strain, Sty1 nuclear exclusion was severely impaired and persisted in the nucleus up to 60 min after stress exposure.

Sustained nuclear localization of Sty1 has been described in the *pyp2∆* mutant and has been associated with rapid re-localization of Sty1 to the nucleus due to low phosphatase activity in the cytoplasm^23^. In our study, although the phosphatases were active, the sustained activity of Wis1 could re-phosphorylate Sty1 and induce its relocation to the nucleus.

Our results describe the first negative feedback regulation of the MAPK pathway through phosphorylation of the Wis1 MAPKK in yeast. In mammals, although no MAPKK has been described as MK2 substrate so far, research also indicates that the inhibitory phosphorylation of MAPKKs catalyzed by MAPKs occurs^26,27^. Furthermore, Srk1 and MK2, although they have differences, they share functional characteristics. They regulate the cell cycle through Cdc25, form a complex with MAPK p38, and are degraded to terminate or modulate the stress response^28–30^. Our preliminary data also suggest a conservation of the negative feedback regulation of p38 by MK2 in mammalian cells. Further studies are however needed to confirm this idea.

**Figure 7:**
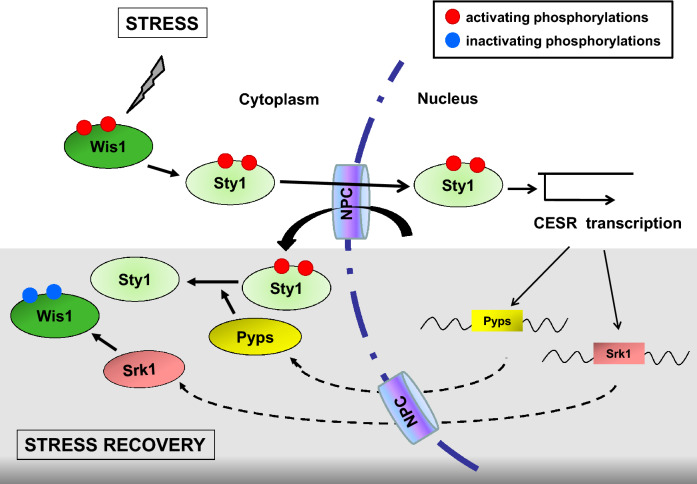
Proposed model for the negative feedback function of Srk1 in the MAPK pathway stress recovery. In response to stress, activation of the Wis1-Sty1 axis induces the transcription of the *CESR –Core Environmental Stress Response-* genes, including *srk1* and *pyp1/pyp2*. The presence of the new Srk1 in the cytoplasm phosphorylates Wis1 in the Sty1-binding domain, which disrupts activation of the Wis1-Sty1 axis and allows stress phosphatases, Pyp1 and Pyp2, to inactivate Sty1.

In summary, we can state that Srk1 has two functions in response to stress. The first,involves the phosphorylation and activation of Srk1 by Sty1 in response to stress, which results in the transient inactivation of the cell cycle (mediated by Cdc25 phophorylation)^18^. In addition, phosphorylation of Srk1 by Sty1 also results in Srk1 degradation. The delay between these two events offers a window of opportunity where the cell can elicit transcriptional response to stress^18,20^. The second function of Srk1 synthesized de novo in response to stress^20^, concerns its role inhibiting the MAPK cascade by phosphorylating Wis1 in Thr225 essentially, thereby disrupting the association between MAPKK Wis1 and MAPK Sty1 (Fig. 7). This prevents Wis1 from reactivating Sty1, and in turn, allows the dephosphorylation and inactivation of Sty1 by the Pyp1 and Pyp2 phosphatases, ultimately silencing the pathway.

## Material and methods

### Fission yeast strain, growth conditions, and reagents

The *S. pombe* strains used in this work are listed in Supplementary information Table S1. They were routinely grown by shaking at 30 °C in rich yeast extract plus supplement (YES) medium or Edinburgh minimal (EMM2) medium with 2% glucose, and supplemented with adenine, leucine, histidine, uracil, and lysine (225 mg/L) (Moreno and Nurse, 1991). In the stress experiments, log-phase cultures (OD_600_ of 0.5; ⁓10^6^ cells/mL) were supplemented with either KCl or H_2_O_2_. At different times, 50 mL of culture cells were harvested by filtration, washed with cold 20% TCA or PBS buffer, as required, and immediately frozen as yeast pellets for further analysis. Transformants expressing constructs from pREP3X-based plasmid were grown in liquid EMM2 medium with thiamine (5 mg/L) and transferred to EMM2 lacking thiamine for at least 20 h.

### Side-directed mutagenesis

The plasmids created and the oligonucleotides used are listed in Supplementary information Tables S2 and S3, respectively. Strains expressing different genomic fusions in multiple genetic backgrounds were constructed either by transformation or after random spore analysis of appropriate crosses in sporulation agar medium.

### Plasmid and strain construction

We constructed the pREP81-*wis1(6HisHA)* and pREP81-*wis1-ED(6HisHA)* plasmids containing the entire *wis1* and *wis1-5A* ORF under the inducible promoter *nmt81*^31^, fused to two hemagglutinin (HA) epitopes and a hexahistidine tag at the C-terminal end, by PCR amplification from genomic DNA. The *srk1*, *wis1*, *wis1-4A*, *wis1-5A*, *wis1-ED*, *wis1-K349R*, *wis1-4A-K349R*, and *wis1-5A-K349R* genes were cloned into the bacterial expression plasmid pGEX-KG, which allows the expression of GST-fused proteins in *E. coli*. The *srk1:kanMX6* strain was constructed by PCR amplification with *srk1*-specific primers and the p*FA6a-kanMX6* plasmid template. Recombinants with the strains in Supplementary information Table S1 were selected based on their ability to grow on G418/geneticin (Invitrogen) and Ura + (Sigma), as described previously^32^.

### Expression and purification of GST fusion proteins

The GST fusion proteins were expressed in *E. coli* BL21 (D3) pLys cells. Log-phase cultures grown at 37 °C were treated with 0.5 mM isopropyl-s-thio-D-galactoside and grown for additional 4 h at 30 °C. Cells were harvested and lysed in NETN buffer (20 mM Tris–HCl [pH 8.0], 100 mM NaCl, 1 mM EDTA, 0.5% NP-40, 20 μg/mL aprotinin, 40 μg/mL leupeptin, 20 μg/mL pepstatin A, and 1 mM PMSF). GST proteins were purified by adsorption to glutathione-Sepharose beads (Amersham-Pharmacia), according to the manufacturer's instructions, and proteins were eluted into 50 mM Tris (pH 8.5), 100 mM NaCl, 10 mM glutathione, and 2 mM DTT. Untagged Srk1 protein was cleaved from GST fusion proteins bound to GST Sepharose beads by digestion with thrombin (10 units/mg protein) (Amersham-Biosciences).

### Western blotting

We lysed 1 × 10^8^ cells in buffer (150 mM NaCl, 50 mM Tris–HCl [pH 8.0], 5 mM EDTA, 0.1% Triton X-100, 10% glycerol, 50 mM NaF, 1 mM PMSF, 1 mM NaVO_4_, 5 μg/mL aprotinin, and 5 μg/mL leupeptin). Proteins were resolved by SDS-PAGE and analyzed by western blotting. Blot membranes were cut prior antibodies incubation, The following primary antibodies were used: monoclonal anti-HA (12CA5) (Roche) (1/1000), monoclonal anti-myc (9E10) (Upstate) (1/1000), polyclonal anti-HA (Upstate) (1/2000), anti-polyclonal anti-phospho Tyr 4G10 (Millipore) (1/1000), monoclonal anti-HOG (Santa Cruz) (1/1000), and polyclonal anti-phospho p38 (Cell Signaling) (1/1000). Horseradish peroxidase-conjugated anti-mouse or anti-mouse antibodies (Bio-Rad) were used as secondary antibodies. Membranes were developed by enhanced chemiluminescence (ECL kit, Amersham-Pharmacia).

### In vitro kinase assay

GST fusion proteins were incubated in the kinase buffer (50 mM Tris–HCl [pH 8.0], 10 mM MgCl_2_, 5 mM β-Mercaptoethanol, 50 μM ATP, and 0.1 μCi/μL [γ-^32^P]ATP) and 350 nM of either GST-Srk1 or Srk1 for 30 min at 30 °C. Labeled proteins were resolved by SDS-PAGE and detected by autoradiograph.

### Plate assays of stress sensitivity for growth

Wild-type and mutant *S. pombe* strains were grown in YES liquid medium to an OD600 of 0.4–0.3, and appropriate decimal dilutions were spotted per triplicate on YES solid plates or in the same medium supplemented with varying concentrations of the stress agent. Plates were incubated at 30 °C for 3 days and then photographed. All assays were repeated at least three times with similar results. Representative experiments are shown in the corresponding figures.

### Immunofluorescence microscopy

Immunofluorescence microscopy was performed by methanol fixation, as described elsewhere (http://pingu.salk.edu/forsburg/lab.html#info). Rabbit affinity-purified anti-HA antibodies (Sigma, H6908) (1/500) were detected with Alexa Fluor 594® conjugated goat anti-rabbit antibodies (Molecular Probes-Alexa). Cells were examined with a confocal laser-scanning microscope (JCS-NT, Leica).

### Statistical analysis

All experiment were done at least three times, Statistical analyses were performed with Prism5.

## Supplementary Information

Supplementary Information.

## Data Availability

The datasets supporting the current study are available from the corresponding author on request.
