# Supplementary material for: A new negative feedback mechanism for MAPK pathway inactivation through Srk1 MAPKAP kinase
Source: Sci Rep. 2022 Nov 14;12:19501. doi: 10.1038/s41598-022-23970-8 (PMC9663701; doi:10.1038/s41598-022-23970-8)

A new negative feedback mechanism for MAPK pathway inactivation through Srk1 MAPKAP kinase.

Maribel Marquina[ad]*, Eva Lambea[a1]*, Mercé Carmona[c], Marta Sánchez-Marinas[a2], Sandra López-Aviles[b], José Ayte[c], Elena Hidalgo[c] and Rosa Aligue[a+]

Supplementary Information

Supplementary Fig. S1

Wis1

```
MSSPNNQPLS CSLRQLSISP TAPPGDVGTP GSLLSLSSSS
SSNTDSSGSS LGSLSLNSNS SGSDNDSKVS SPSREIPSDP
PLPRAVPTVR LGRSTSSRSR NSLNLDMKDP SEKPRRSLPT
AAGQNNIGSP PTPPGPFPGG LSTDIQEKLK AFHASRSKSM
PEVVNKISSP TTPIVGMGQR GSYPLPNSQL AGRLSNSPVK
SPNMPESGLA KSLAAARNPL LNRPTSFNRQ TRIRRAPPGK
LDLSNSNPTS PVSPSSMASR RGLNIPPTLK QAVSETPFST
FSDILDAKSG TLNFKNKAVL NSEGVNFSSG SSFRINMSEI
IKLEELGKGN YGVVYKALHQ PTGVTMALKE IRLSLEEATF
NQIIMELDIL HKAVSPYIVD FYGAFFVEGS VFICMEYMDA
GSMDKLYAGG IKDEGVLART AYAVVQGLKT LKEEHNIIHR
DVKPTNVLVN SNGQVKLCDF GVSGNLVASI SKTNIGCQSY
MAPERIRVGG PTNGVLTYTV QADVWSLGLT ILEMALGAYP
YPPESYTSIF AQLSAICDGD PPSLPDSFSP EARDFVNKCL
NKNPSLRPDY HELANHPWLL KYQNADVDMA SWAKGALKEK
GEKRS
```

Fig. S1. Wis1 protein sequence.
Srk1 consensus phosphorylation sites in Wis1 (shown in blue).
New Srk1 non consensus phopshorylation site in Wis1 (shown in red).
Wis1 accession number X62631.1 and SPBC409.07c.

Supplementary Table S1

Strain	Genotype	Origin
**RA2502**	*h- sty1::ura4+ leu1-32 ura4-D18*	Lab stock
**RA0770**	*h- wis1::ura4+ leu1-32 ura4-D18*	Lab stock
**RA1102**	*h- srk1::kanMX6 leu1-32 ura4-D18*	Lab stock
**RA0772**	*h- atf1::ura4+ leu1-32 ura4-D18*	Lab stock
**RA1449**	*h- srk1::kanMX6 atf1::ura4+ leu1-32 ura4-D18*	Lab stock
**RA1380**	*h- srk1:HA:kanMX6 leu1-32 ura4-D18*	Lab stock
**RA3917**	*h- srk1-K153A:kanMX6 leu1-32 ura4-D18*	Lab stock
**RA1843**	*h- pyp1:12myc:ura4+ leu1-32 ura4-D18*	Dr. P. Russell
**RA1920**	*h- pyp1:12myc:ura4+ srk1::kanMX6 leu1-32 ura4-D18*	This study
**RA1844**	*h- pyp2:12myc:ura4+ leu1-32 ura4-D18*	Dr. P. Russell
**RA1922**	*h- pyp2:12myc:ura4+ srk1::kanMX6 leu1-32 ura4-D18*	This study
**RA1423**	*h+ pyp1::leu2 leu1-32 ura4-D18*	Dr. J. Millar
**RA1424**	*h+ pyp2::leu2 leu1-32 ura4-D18 ade6-704*	Dr. J. Millar
**RA1866**	*h- pyp1::ura4+ srk1::kanMX6 leu1-32 ura4-D18*	This study
**RA1919**	*h- pyp2::ura4+ srk1::kanMX6 leu1-32 ura4-D18*	This study
**RA1973**	*h+ wis1::kanMX6 leu1-32 ura4-D18*	Lab stock
RA2152	*h- wis1-DD:ura4+ leu1-32*	lab stock
**RA1425**	*h- wis1:12myc::ura4+ leu1-32 ura4-D18*	Dr. P. Russell
**RA1563**	*h- wis1:12myc::ura4+ srk1::kanMX6 leu1-32 ura4-D18*	This study
**RA0705**	*h- sty1:6His-HA:ura4* $^{+}$ *leu1-32 ura4-D18*	Dr. J. Millar
**RA3138**	*wis1:HA:kanMX6 leu1-32 ura4-D18*	Lab stock
**RA4025**	*wis1:TE/SD(ED)-HA:kanMX6 leu1-32 ura4-D18*	This study
**RA4024**	*h+ wis1:5A-HA:kanMX6 leu1-32 ura4-D18*	This study
**RA1579**	*h- cdc25-9A:ura4+ leu1-32*	lab stock

Table S1. Strain used.

Supplementary Table S2

Plasmid	Sites	Origin
pGEX-KG-*srk1*	NdeI-NotI	Lab stock
pGEX-KG-*srk1K153A*	NdeI-NotI	Lab stock
pGEX-KG-*sty1*	NdeI-NotI	Dra. E. Hidalgo
pGEX-KG-*sty1KA*	NdeI-NotI	Dra. E. Hidalgo
pGEX-KG-*atf1*	NdeI-NotI	Dr. J. Millar
pREP41-*srk1-HA*	NdeI-NotI	Lab stock
pREP1-*srk1-HA*	NdeI-NotI	Lab stock
pREP1-*srk1-K153A-HA*	NdeI-NotI	Lab stock
pGEX-KG-*wis1*	NdeI-NotI	This study
pGEX-KG-*wis1-5A*	NdeI-NotI	This study
pGEX-KG-*wis1-T225E/S226D*	NdeI-NotI	This study
pGEX-KG-*wis1-K349R*	NdeI-NotI	This study
pGEX-KG-*wis1-4A-K349R*	NdeI-NotI	This study
pGEX-KG-*wis1-5A-K349R*	NdeI-NotI	This study

Table S2. Plasmids used

Supplementary Table S3

Oligonucleotide	Sequence	Objective
Srk1 fwd	$^{5'}$CACACACATATGCGTTTTAAAAGTATTCAGCAAAATATCGAGGA$^{3'}$	Oligos to clone *srk1 and srk1T463A* genes in pGEX and pREP1 plasmids containing NdeI and NotI sites respectively.
Srk1 rev	$^{5'}$CACACAGCGGCCGCCACTTTTTGTCGATGTCGACGATTATAC$^{3'}$	
Wis1-K349R 5'	$^{5'}$GGTGTCACTATGGCCTTGCGCGAAATTAGGTTGTCC$^{3'}$	Oligos to mutate K349 of *wis1* gene to Arg (in red).
Wis1-K349R 3'	$^{5'}$GGACAACCTAATTTCGCGCAAGGCCATAGTGACACC$^{3'}$	
Wis1-S17A fwd	$^{5'}$CGCGCGCATATGTCTTCTCCAAATAATCAACCCTTGTCTTGCTCATTGAGACAGCTGGCTATTTCTCCTACCGCACCTCCCGG^{3}	Oligos to mutate S17 of *wis1* gene to Ala (in red) containing NdeI and NotI sites respectively.
Wis1 rev	$^{5'}$GGCGCTCTTAAAGAGAAAGGTGAAAAAAGAAGCTGCGGCCGCCGCGCG$^{3'}$	
Wis1-S96A fwd	$^{5'}$GCCTACGGTCAGACTTGGCAGATCTACGGCCAGTCGGAGTCGTAACTCTCTTAACCTTGAC$^{3'}$	Oligos to mutate S96 of *wis1* gene to Ala (in red).
Wis1-S96A rev	$^{5'}$GTCAAGGTTAAGAGAGTTACGACTCCGACTGGCCGTAGATCTGCCAAGTCTGACCGTAGGC$^{3'}$	
Wis1-S159A fwd	$^{5'}$GGCCTTCCATGCATCTAGATCAAAAGCAATGCCGGAAGTAGTCAACAAGATCAG$^{3'}$	Oligos to mutate S159 of *wis1* gene to Ala (in red).
Wis1-S159A rev	$^{5'}$CTGATCTTGTTGACTACTTCCGGCATTCGTTTTGATCTAGATGCATGGAAGGCC$^{3'}$	
Wis1-S226A fwd	$^{5'}$GGAATCCTTTACTCAACCGTCCAACGGCCTTCAATCGACAAACGAGAATCCGTCG$^{3'}$	Oligos to mutate T226 of *wis1* gene to Ala (in red).
Wis1-S226A rev	$^{5'}$CGACGGATTCTCGTTTGTCGATTGAAGGCCGTTGGACGGTTGAGTAAAGGATTCC$^{3'}$	
Wis1-T225A-S226A fwd	$^{5'}$GGAATCCTTTACTCAACCGTCCAGCGGCCTTCAATCGACAAACGAGAATCCGTCG$^{3'}$	Oligos to mutate T225 and S226 of *wis1* gene to Ala (in red).
Wis1-T225A-S226A rev	$^{5'}$CGACGGATTCTCGTTTGTCGATTGAAGGCCGCTGGACGGTTGAGTAAAGGATTCC$^{3'}$	
Wis1-T225E -S226D fwd	$^{5'}$GGAATCCTTTACTCAACCGTCCAGAGGATTTCAATCGACAAACGAGAATCCGTCG$^{3'}$	Oligos to mutate T225 to Glu and T226 to Asp of wis1 gene (in red).
Wis1-T225E-S226D rev	$^{5'}$CGACGGATTCTCGTTTGTCGATTGAAATCCTCTGGACGGTTGAGTAAAGGATTCC$^{3'}$	

Table S3. Oligonucleotides used

Supplementary Original western blot and kinase assays images

Fig. 1a

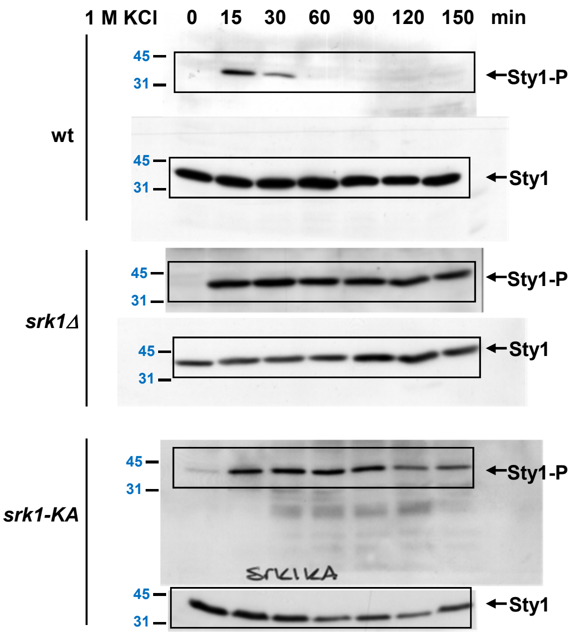

Fig. 1b

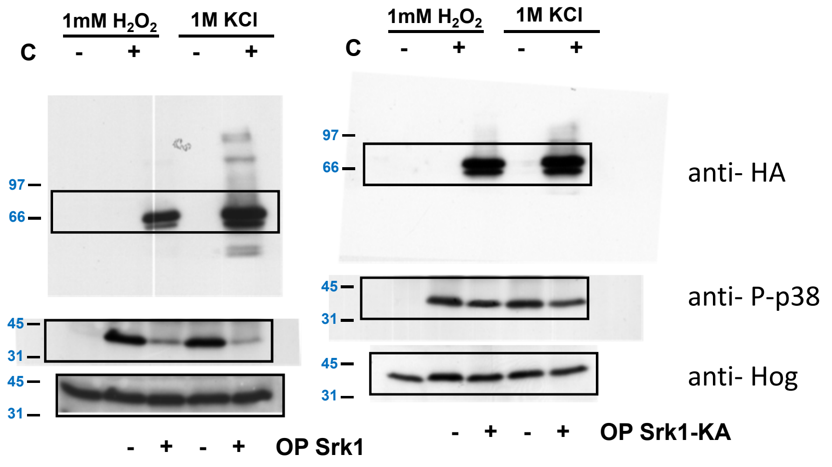

Fig. 1c

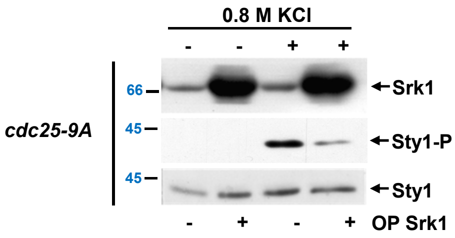

Fig. 1c (replicate)

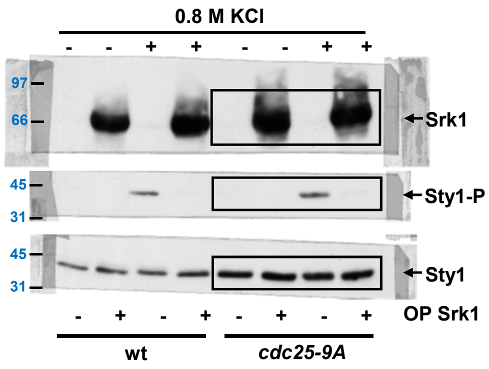

Fig. 1d

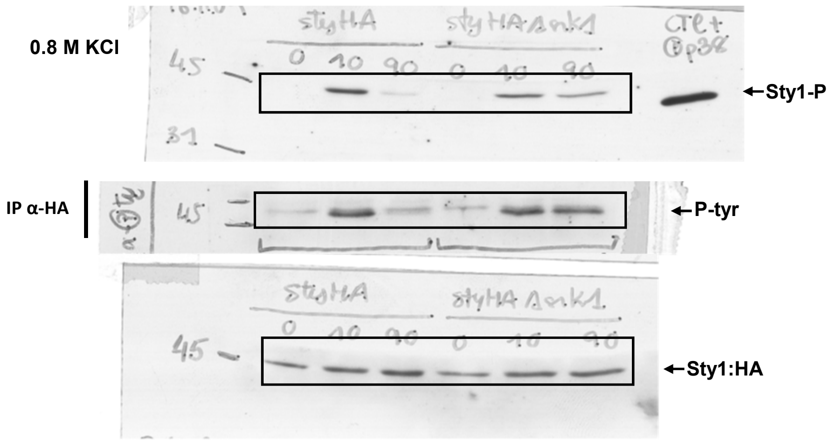

Fig. 2a

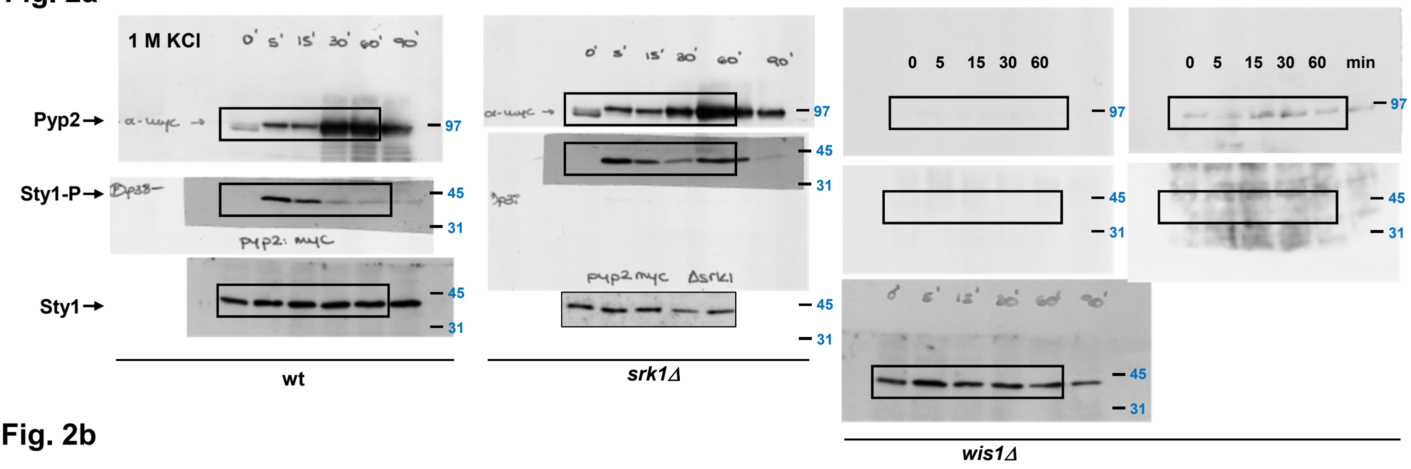

Fig. 2b

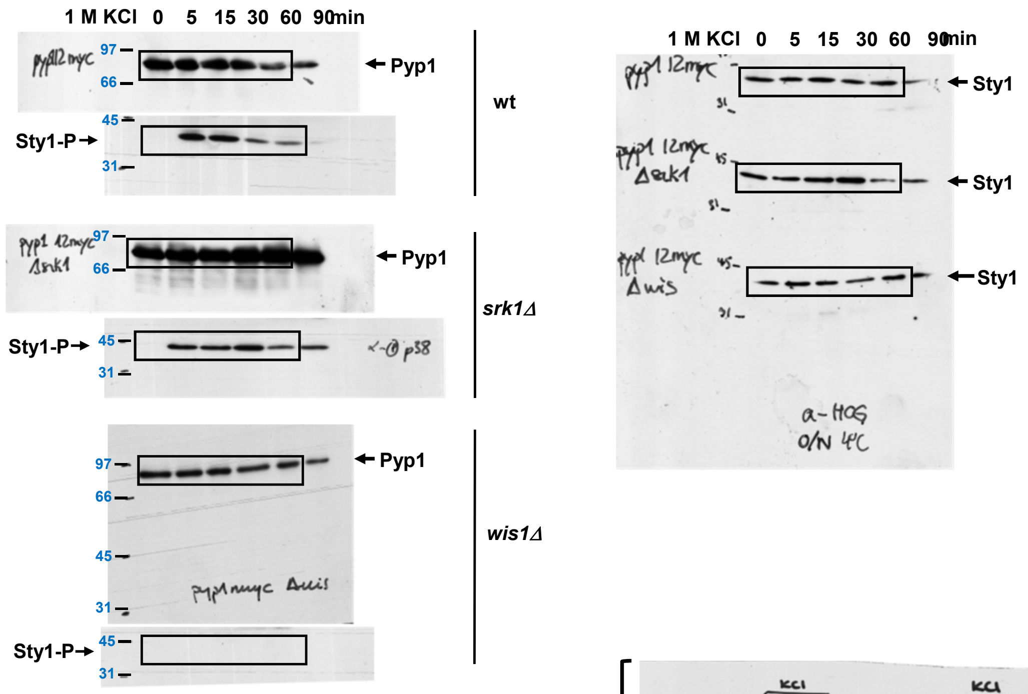

Fig. 2c

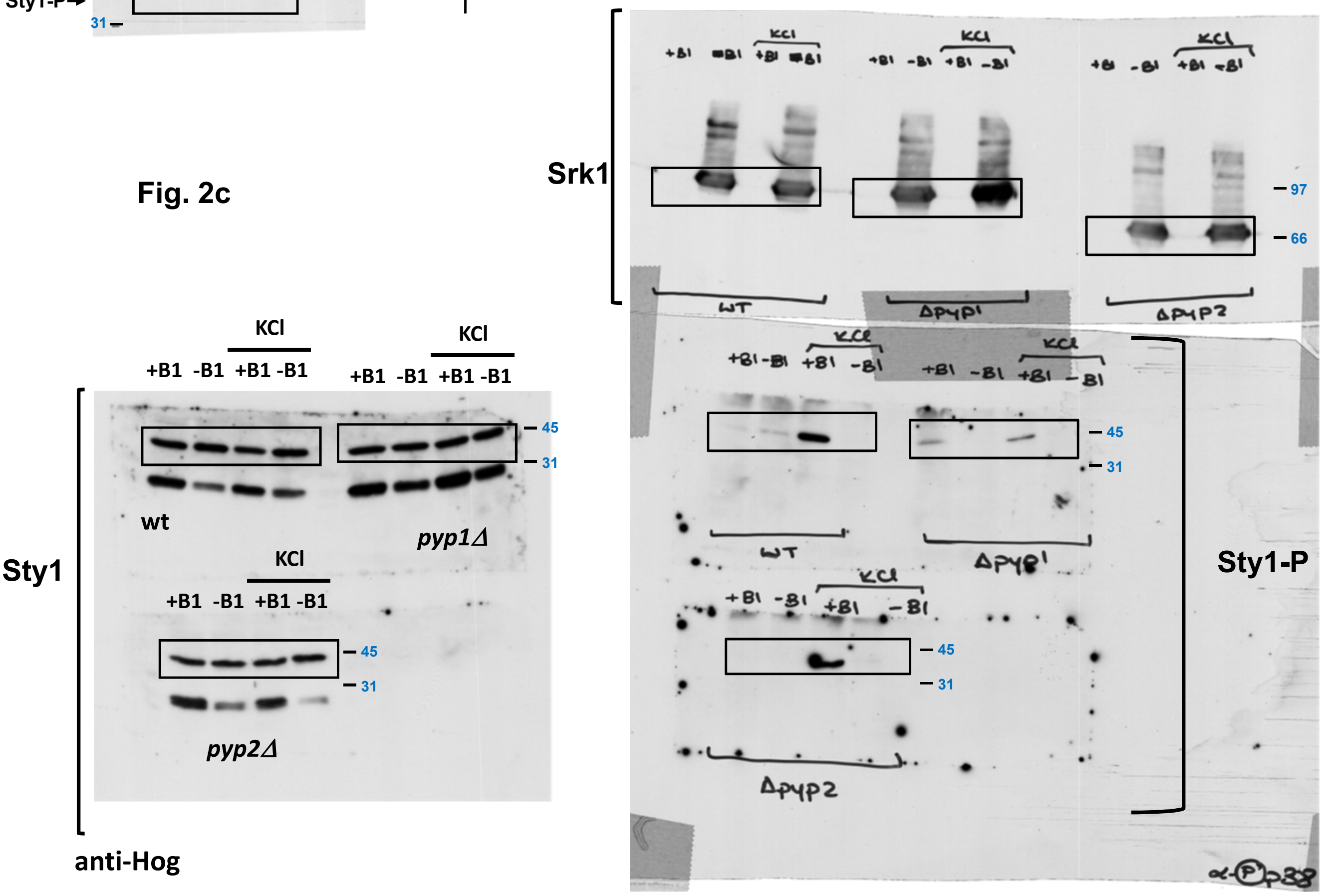

Fig. 3a

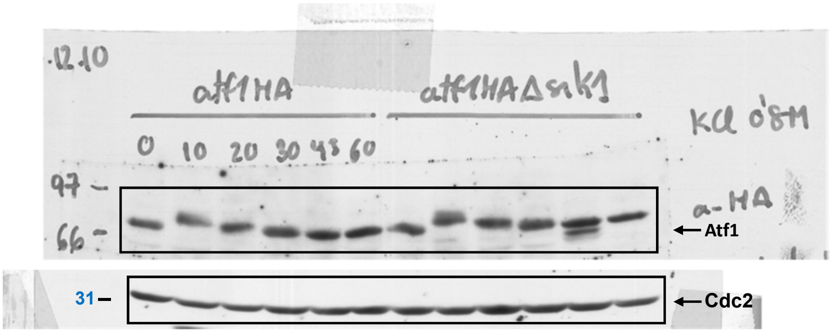

Fig. 3b

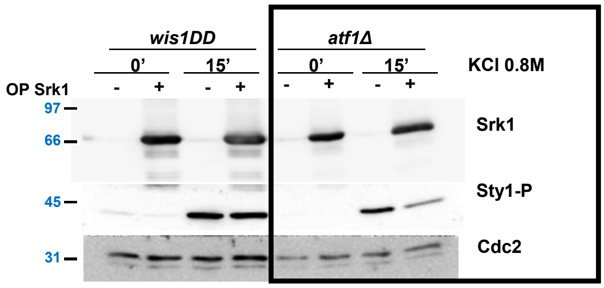

	1	2	3	4	5	6	7	8
p-p38/cdc2	0,04	0,02	1,96	1,03			1,64	0,51

Fig. 5a

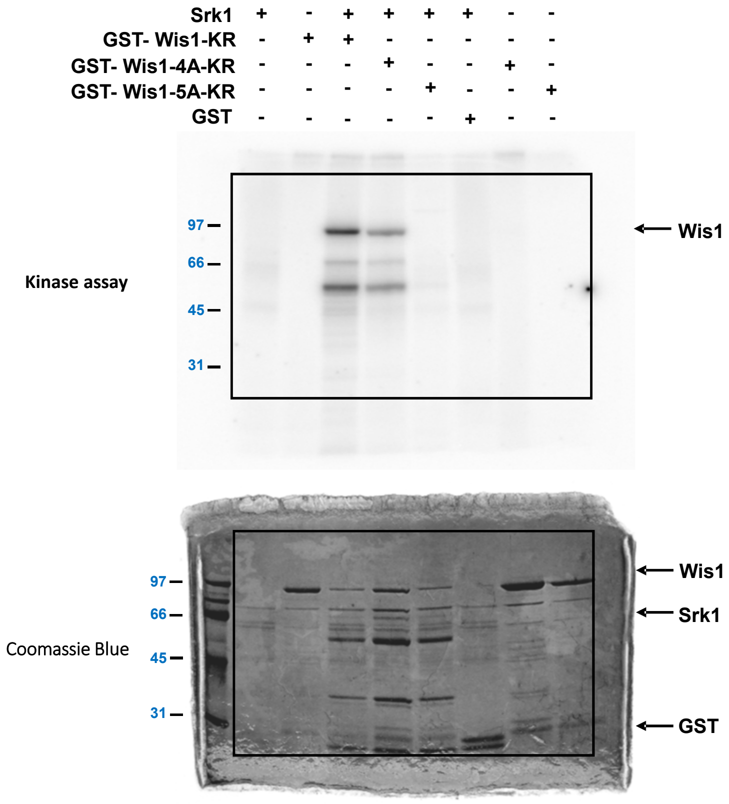

Fig. 5a (phosphorylation of Wis1 and Wis1-4A, replicate)

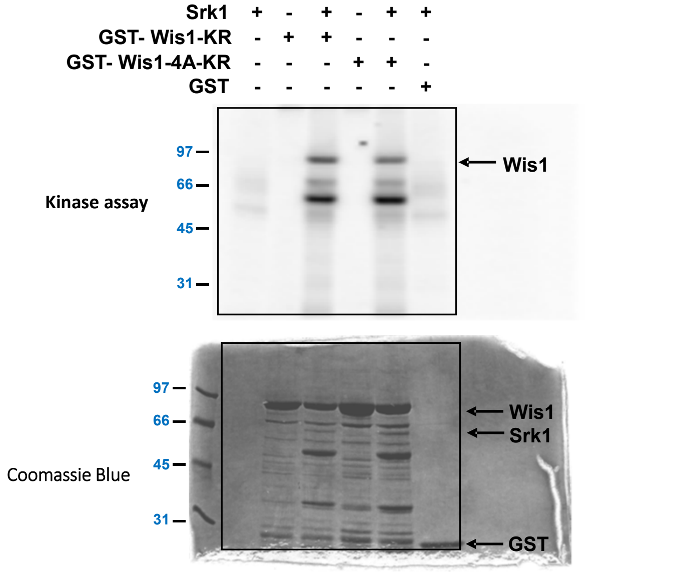

Fig. 6a

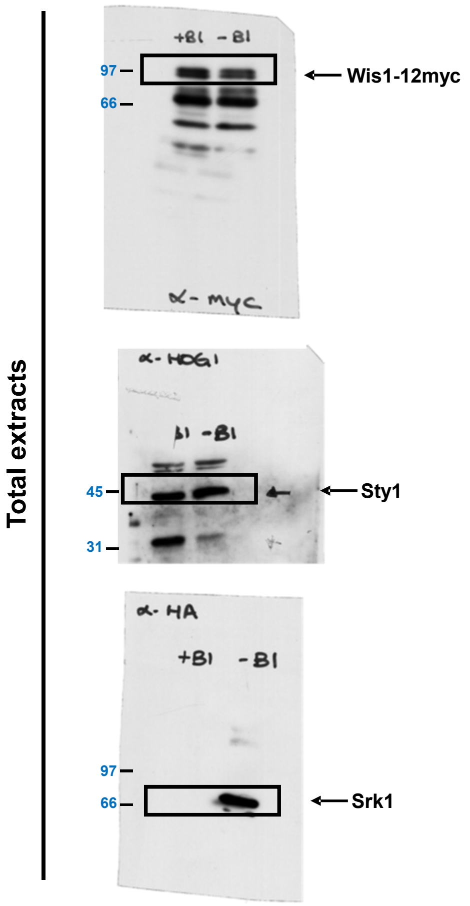

Fig. 6b

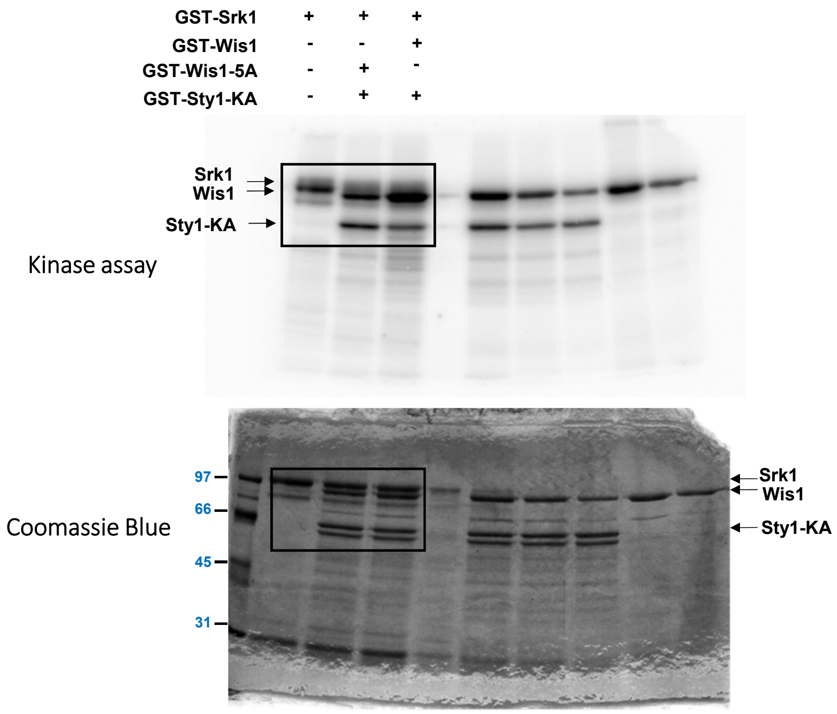

Fig. 6c

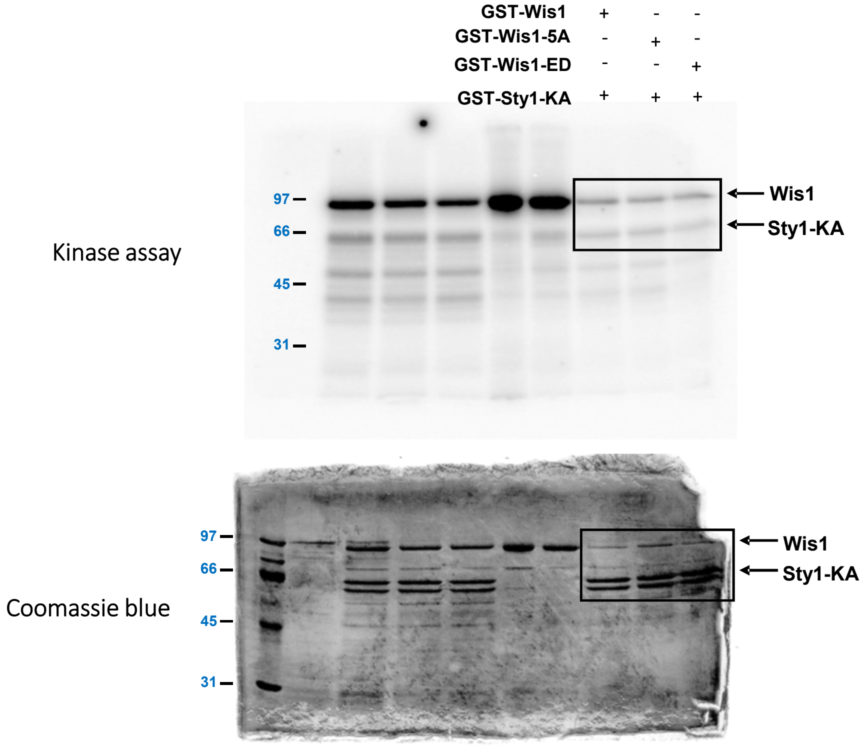

Supplement: Supplementary file 1 — Supplementary Information. [file 41598_2022_23970_MOESM1_ESM.pdf]
